# Mini Review Therapeutic Strategies Targeting for Biofilm and Bone Infections

**DOI:** 10.3389/fmicb.2022.936285

**Published:** 2022-06-14

**Authors:** Shizhou Wu, Binjie Wu, Yunjie Liu, Shu Deng, Lei Lei, Hui Zhang

**Affiliations:** ^1^Department of Orthopedic Surgery, West China Hospital, Sichuan University, Chengdu, China; ^2^West China School of Public Health, Sichuan University, Chengdu, China; ^3^Boston University Henry M. Goldman School of Dental Medicine, Boston, MA, United States; ^4^West China Hospital of Stomatology, Sichuan University, Chengdu, China

**Keywords:** biofilm, bone infections, bone-targeted therapy, immunity homeostasis, microorganisms, *Staphylococcus aureus*

## Abstract

Bone infection results in a complex inflammatory response and bone destruction. A broad spectrum of bacterial species has been involved for jaw osteomyelitis, hematogenous osteomyelitis, vertebral osteomyelitis or diabetes mellitus, such as *Staphylococcus aureus* (*S. aureus*), coagulase-negative Staphylococcus species, and aerobic gram-negative bacilli. *S. aureus* is the major pathogenic bacterium for osteomyelitis, which results in a complex inflammatory response and bone destruction. Although various antibiotics have been applied for bone infection, the emergence of drug resistance and biofilm formation significantly decrease the effectiveness of those agents. In combination with gram-positive aerobes, gram-negative aerobes and anaerobes functionally equivalent pathogroups interact synergistically, developing as pathogenic biofilms and causing recurrent infections. The adhesion of biofilms to bone promotes bone destruction and protects bacteria from antimicrobial agent stress and host immune system infiltration. Moreover, bone is characterized by low permeability and reduced blood flow, further hindering the therapeutic effect for bone infections. To minimize systemic toxicity and enhance antibacterial effectiveness, therapeutic strategies targeting on biofilm and bone infection can serve as a promising modality. Herein, we focus on biofilm and bone infection eradication with targeting therapeutic strategies. We summarize recent targeting moieties on biofilm and bone infection with peptide-, nucleic acid-, bacteriophage-, CaP- and turnover homeostasis-based strategies. The antibacterial and antibiofilm mechanisms of those therapeutic strategies include increasing antibacterial agents’ accumulation by bone specific affinity, specific recognition of phage-bacteria, inhibition biofilm formation in transcription level. As chronic inflammation induced by infection can trigger osteoclast activation and inhibit osteoblast functioning, we additionally expand the potential applications of turnover homeostasis-based therapeutic strategies on biofilm or infection related immunity homeostasis for host-bacteria. Based on this review, we expect to provide useful insights of targeting therapeutic efficacy for biofilm and bone infection eradication.

## Introduction

**FIGURE 1:**
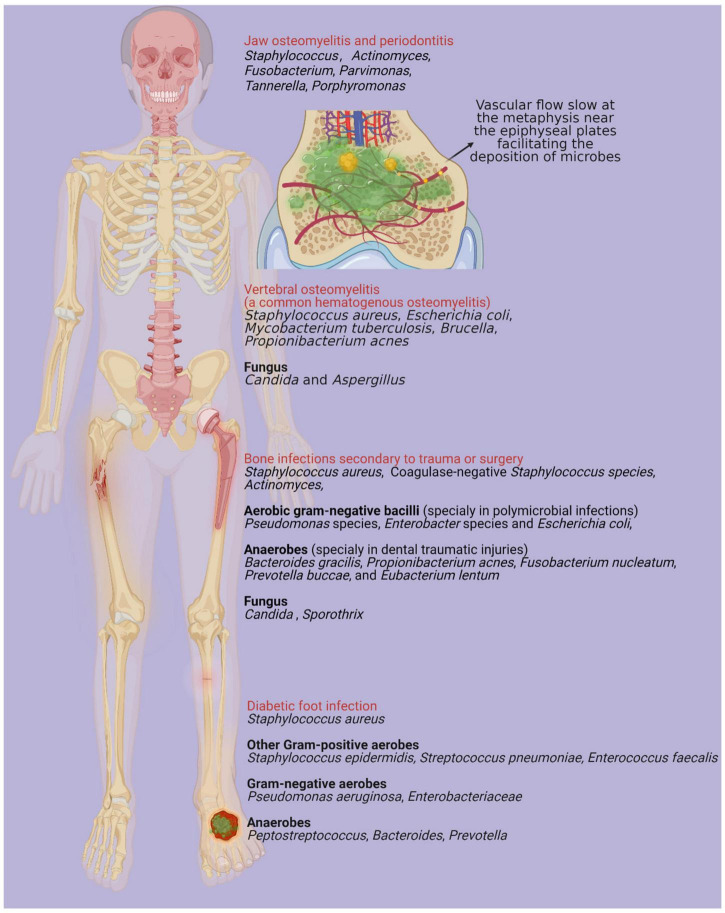
There are various microorganisms associated with different types of bone infections, mainly including jaw osteomyelitis, periodontitis, bone infections secondary to trauma or surgery, diabetic foot infection and hematogenous osteomyelitis (Lew and Waldvogel, 2004). Vascular flow slows at the metaphysis near the epiphyseal plates facilitating the deposition of microbes and vertebral osteomyelitis is one of most common types in hematogenous osteomyelitis (Maamari et al., 2022). Created with BioRender.com.

Bone infection is an inflammatory process characterized by microorganism invasion, which results in localized bone loss and destruction (Lew and Waldvogel, 2004; Chen et al., 2021). A broad spectrum of bacterial species has been isolated in cases of bone infection diseases. Although various antibiotics have been applied for bone infection, the emergence of drug resistance and biofilm formation significantly decrease the effectiveness of those agents. Biofilms are matrices comprising polysaccharides, proteins, extracellular DNA (eDNA) and host components that are encased within bacterial communities (Hall-Stoodley et al., 2004; Lei et al., 2021). The adhesion of biofilms to bone promotes bone destruction and protects bacteria from antimicrobial agent stress and host immune system infiltration (Sanchez et al., 2013). In jaw osteomyelitis and periodontitis, multiple species, including species of *Actinomyces, Fusobacterium, Parvimonas, Tannerella, Porphyromonas*, and *Staphylococcus*, are synergistically involved (Gaetti-Jardim et al., 2010; Settem et al., 2012; Chen et al., 2021). Moreover, several species have been identified in bone infections secondary to trauma, surgery or insertion of a joint prosthesis, including *Staphylococcus aureus*, coagulase-negative Staphylococcus species, and aerobic gram-negative bacilli (*Pseudomonas* species, *Enterobacter* species and *Escherichia coli*). Those particularly isolated in polymicrobial infections include anaerobes (such as *Bacteroides gracilis*, *Propionibacterium acnes*, *Fusobacterium nucleatum*, *Prevotella buccae*, and *Eubacterium lentum*), and those particularly isolated in traumatic dental injuries include species of *Actinomyces*, *Candida*, and *Sporothrix* (Lew and Waldvogel, 2004; Schmitt, 2017; Fouad, 2019). Hematogenous osteomyelitis (AHO) is usually monomicrobial. *S. aureus* is a predominant bacterium in AHO and can also combine with other microorganisms, including *Streptococcus*, *Salmonella*, *Enterobacter cloacae*, *Pseudomonas aeruginosa*, and *Bartonella henselae* (McNeil et al., 2016; Schmitt, 2017; McNeil, 2020). Microorganisms in blood are in slow flow, particularly at the vascular loops. Metaphysis near the epiphyseal plates distributed with vascular loops facilitates the deposition of microbes (Calhoun et al., 2009). Vertebral osteomyelitis is also commonly induced by the hematogenous deposition of microbes in vertebral bodies (Maamari et al., 2022). *S. aureus* is the most common pathogen in the setting of vertebral osteomyelitis, which is particularly assumed to be associated with *S. aureus* bacteremia (Berbari et al., 2015). Alternative pathogens include *Escherichia coli*, *Mycobacterium tuberculosis*, *Brucella*, *Propionibacterium acnes, Candida*, and *Aspergillus*, which are involved in spinal surgery, immunocompromised patients and intravenous catheters (Berbari et al., 2015). Diabetes mellitus, as a worldwide public health threat, leads to significant bone and soft-tissue ischemia, peripheral neuropathy and immunocompromise. All these pathological changes subsequently result in skin ulceration and diabetic foot infections (Jneid et al., 2017). By microbiota analysis, diabetic cutaneous tissue presents an increase in *S. aureus* compared with non-diabetic cutaneous tissue (Redel et al., 2013). A decrease in bacterial diversity and an increase in opportunistic pathogens have been identified in infective diabetic cutaneous tissue compared with contralateral intact skin (Gontcharova et al., 2010; Dunyach-Remy et al., 2016). In combination with gram-positive aerobes (*Staphylococcus epidermidis, Streptococcus pneumoniae*, *Enterococcus faecalis*, etc.), gram-negative aerobes (*Pseudomonas aeruginosa*, *Enterobacteriaceae*, etc.), and anaerobes (*Peptostreptococcus* spp., *Bacteroides* spp., *Prevotella* spp., etc.), functionally equivalent pathogroups interact synergistically, developing as pathogenic biofilms and causing recurrent diabetic foot infections (Claros et al., 2013; Lipsky et al., 2013; Jneid et al., 2017). The above etiologies of microbial distribution and pathological mechanism are mapped in Figure 1.

**FIGURE 2:**
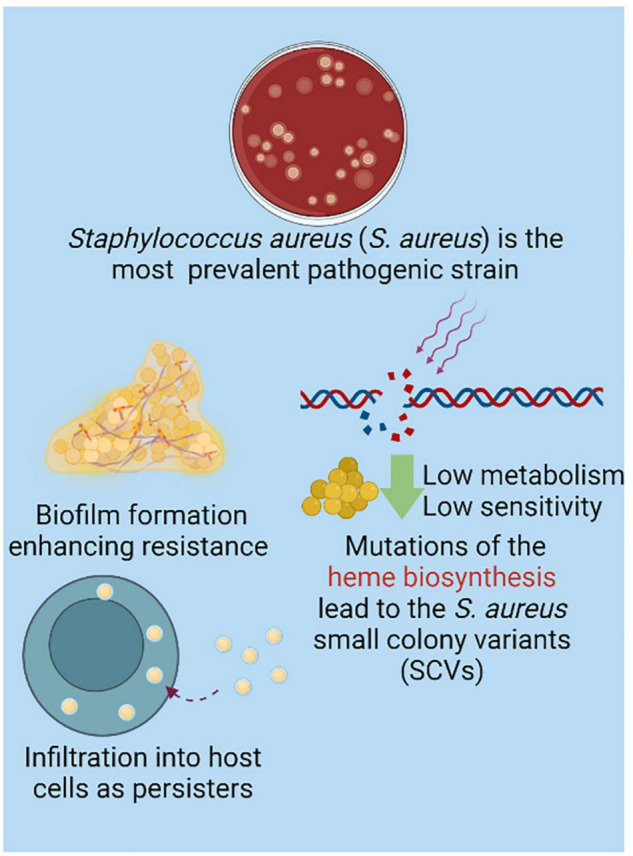
Although species contribute to bone infection is in diversity, *Staphylococcus* aureus (*S. aureus*) is the most prevalent isolated strain (Wright and Nair, 2010). The biofilm formation significantly enhances the resistance of strains (Sanchez et al., 2013). With mutation of heme biosynthesis, small colony variant of *S. aureus* (SCV) is charactered by low metabolism which resulted in low sensitivity to the conventional antibacterial strategy. Interestingly, the intracellular SCV in host cells are causing the recurrent and refractory infective locus as persisters, which further provide the resistance of *S. aureus* (Proctor, 2019). Created with BioRender.com.

*S. aureus* is the most prevalent bacterial species that contributes to bone infections (Wright and Nair, 2010). Many mechanisms have been applied to enhance *S. aureus* resistance (Figure 2). Bone, as a highly mineralized tissue, is characterized by low permeability and reduced blood flow, further increasing the difficulty of treatment for bone infections (Shi et al., 2020). Notably, there are a group of clones derived from mutations in the electron transport pathway that impair the growth of bacterial cells, resulting in cells that are smaller in size. These so-called small colony variants (SCVs) particularly adapt to invading and persisting within host cells (Proctor et al., 2006; Proctor, 2019). This internalized *S. aureus* can survive in a dormant state, enabling bacterial escape from antimicrobials and host immune responses. Taken together, SCV cells do not respond to clinical treatment effectively and are related to latent or recurrent infections, representing a troublesome problem in the management of *Staphylococcal* bone disease (Proctor et al., 2006). As conservative antibiotics should penetrate through the rigid bone structure, biofilm and even host cells to maintain a local sufficient therapeutic concentration, bone infection treatment can be challenging. To improve therapeutic effects and minimize systematic toxicity, infective bone locus-targeted management with high efficacy on biofilm bacteria could serve as a promising modality (Zimmerli and Sendi, 2017). Herein, we summarize recent targeting moieties on biofilm and bone infection with peptide-, nucleic acid-, bacteriophage-, CaP- and turnover homeostasis-based strategies.

## Peptide-Based Bone-Targeted Therapeutic Strategy Negatively Regulate Biofilm Development and Maintenance

**FIGURE 3:**
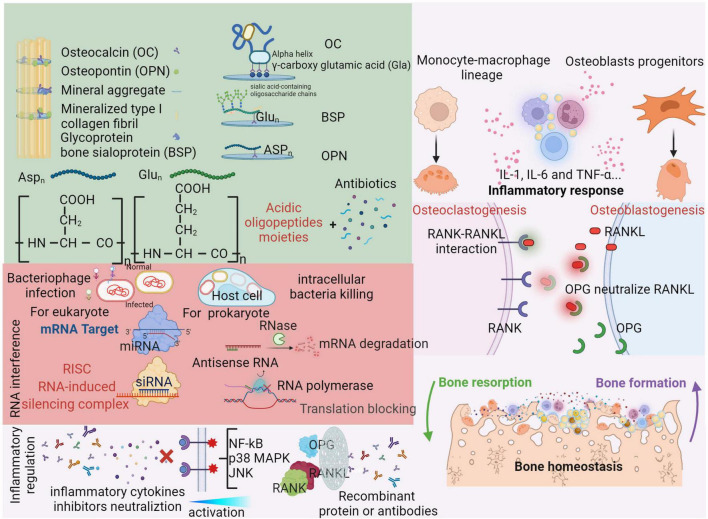
Organic compounds are also essential for bone matrix. Besides collagenous proteins, non-collagenous proteins (OC, BSP, and OPN) can strongly bind to Ca^2+^ or the mineral surfaces of bone with repetitive acidic amino acids moieties (aspartate, Asp_*n*_ and glutamic, Glu_*n*_) (Takahashi-Nishioka et al., 2008). Through three neighboring γ-carboxy glutamic acid (Gla) residues on the same face of the alpha helix, osteocalcin (OC) can bind with mineral aggregate (Stock, 2015). RNA interference (RNAi) is effectiveness in transcriptive level as a novel administration. For bacterial elimination, antisense RNA can recruit RNase to degrade base-paired mRNA and repel RNA polymerase to block translation (Quemener et al., 2022). Similarly, miRNA and siRNA can form RNA-induced silencing complex intraocularly for eukaryote, which are enable to modulate bone homeostasis (Jinek and Doudna, 2009). Bacteriophages can specifically and continually infect bacterial even in host cells. Therefore, a cocktail of several phages is designed as an infection targeting therapy for osteomyelitis (Chan et al., 2013). Please see above context in red background. In responses to bone infection, the inflammation induced release of various cytokines (IL-1, IL-6, and TNF-α) which can active the nuclear factor kB (NF-kB), p38 mitogen-activated protein kinase (MAPK) and c-Jun N-terminal kinase (JNK) pathways (Wright and Nair, 2010). Subsequently, the expressions of bone turnover proteins (OPG, RANKL and RANK) and cross-talk between osteoclasts and osteoblasts are alternated. RANKL can be released by osteoblasts and bind with RANK to active osteoclasts. As a result, the activity of bone resorption is significantly increased. Also, osteoblasts can release OPG which plays a role on neutralize RANKL as a soluble decoy receptor (Jin et al., 2007). Recently, many inhibitors or recombinant antibodies are developed as an inflammation targeting therapy to alleviate hyperinflammatory status and rebuilt the balance of bone homeostasis (Tang et al., 2020). Please see detail in light red background. Created with BioRender.com.

Bone is uniquely composed of the inorganic compound hydroxyapatite (HA). Therefore, HA could be a promising target for bone-targeting drug designs. Bone matrix proteins, such as osteopontin, the most abundant non-collagenous protein in bone, are observed to strongly bind to Ca^2+^ and the mineral surfaces of bone. Several bone proteins exhibit repetitive sequences of acidic amino acids (aspartate, Asp or glutamic acid, Glu) (Takahashi-Nishioka et al., 2008). These moieties can complement or chelate with calcium ions in the spatial HA orientation, resulting in a strong affinity to the bone (Wang et al., 2005). Thus, acidic oligopeptide moieties are considered candidates for bone-targeting carriers (Figure 3, green background). The different features in bone formation and bone resorption physical chemistry provide a series of clues for developing specific affinity agents. On the bone formation surfaces, osteoblasts are characteristically covered by lowly crystallized hydroxyapatite and amorphous calcium phosphonate. Nevertheless, the physical chemistry of bone resorption surfaces is characterized by osteoclast distribution on highly crystallized hydroxyapatite (Wang et al., 2007). Bone-targeting oligopeptides have an interesting property of selective binding to bone resorption or bone formation surfaces. Asp8 has a stronger affinity for highly crystallized hydroxyapatite than for less crystallized hydroxyapatite, which is beneficial for binding to bone resorption sites and selectively approaching osteoclasts (Liu et al., 2015). For infection treatment, acidic oligopeptides can chemically conjugate with antibiotics, which enhances the bone specificity of antibiotics and limits their systemic toxicity. Yao et al. (2021) reported that eight repeating sequences of aspartate (D-Asp8) could be used as a bone-targeting delivery system with enoxacin-loaded mesoporous silica nanoparticles (MSNs). Enoxacin, as a third-generation fluoroquinolone antibiotic, has not only a bactericidal effect but also an inhibitory effect on osteoclasts (Toro et al., 2012).

Conversely, six repetitive sequences of (AspSerSer)_6_ are favorable for binding to amorphous calcium phosphate and are regarded as a selective targeting moiety for bone formation surfaces (Zhang et al., 2012). To decrease the potential toxic side effects of bone-targeting delivery systems at the tissue level, such as undesirable accumulation in other endothelial cells or lymphocytes near the bone formation surface, a more targeted system at the cellular level is developed to promote bone-targeting delivery efficacy. Sun et al. (2016) identified an osteoblast-targeted peptide sequence as Ser-Asp-Ser-Ser-Asp (SDSSD). The SDSSD peptide binds with Periostin (an osteoblast-specific factor 2, OSF-2) in a ligand-receptor-specific manner, indicating its osteoblast-targeting ability. With angomirs/antagomirs, a class of endogenous non-coding microRNAs (miRNAs) can widely modulate gene expression to participate in maintaining cellular homeostasis. Induced by anti-miR-214, the levels of miR-214 in osteoblasts were significantly decreased, while bone formation was increased (Sun et al., 2016), which could be applied to stimulate inactivated osteoblasts in the infection microenvironment.

Antibiotic resistance in bacteria, attributed to multiple mechanisms, imposes a serious threat to global health. Moreover, SCVs of *S. aureus* are capable of infiltrating different types of host cells, resulting in regional recurrent and chronic bone infections (Kahl et al., 2016). Through the adhesion of surface proteins, fibronectin-binding proteins (FnBP) and activated uptake, the SCVs of *S. aureus* can be located intracellularly in host cells, particularly in osteoblasts, and osteoclasts (Kavanagh et al., 2018). The internalization of SCVs with osteoclasts promotes proinflammatory cytokine (macrophage inflammatory protein, keratinocyte chemoattractant, and granulocyte macrophage colony-stimulating factor) secretion and enhances the bone resorption capacities of osteoclasts (Trouillet-Assant et al., 2015). Additionally, internalized *S. aureus* can induce apoptosis of osteoblasts *via* the TRAIL/caspase pathway, which results in imbalanced bone homeostasis (Tucker et al., 2000; Ning et al., 2011; Widaa et al., 2012). Recent studies have shown that antimicrobial peptides (AMPs) can be released from osteoblasts infected by *S. aureus* for self-defense as a promising and effective strategy to treat osteomyelitis, notably for intracellular *S. aureus* (Zhu et al., 2013; Josse et al., 2015). From this point of view, AMPs present distinct advantages over traditional antibiotics (Browne et al., 2020). AMPs are part of the innate immune system and exhibit broad-spectrum antimicrobial activity against ESKAPE (referring to *Enterococcus faecium*, *S. aureus*, *Klebsiella pneumoniae*, *Acinetobacter baumannii*, *Pseudomonas aeruginosa*, and *Enterobacter* sp.) (Costa et al., 2021).

The direct interaction on the bacterial cell membrane contributes to the main antibacterial mechanism of AMPs. Usually, AMPs are positively charged hydrophobic peptides. Through electrostatic interactions with anionic bacterial membranes, cationic antimicrobial peptides, such as AMPs, promote pore formation and alternate membrane permeability, ultimately causing bacterial cell death (Zhang and Gallo, 2016). AMPs can also translocate through the membrane to reach intracellular targets. The antibiofilm action mechanisms of AMPs were speculated to act by stimulating bacterial surface motility and reducing cell attachment (Bucki et al., 2010). Moreover, by downregulating quorum-sensing systems, AMPs negatively regulate biofilm development and maintenance (Overhage et al., 2008). The stringent stress response (SR) is highly conserved among gram-negative and gram-positive bacteria. With secondary messenger molecule (p) ppGpp medication, SR plays an important role in biofilm development. AMPs can bind to and degrade secondary messengers, acting as antibiofilm peptides (de la Fuente-Nunez et al., 2014; Pletzer and Hancock, 2016). Considering the dispersal of bacterial cells from biofilms, which may cause recurrent infection, antibiofilm AMPs can be applied synergistically with conventional antibiotics for biofilm infection (Reffuveille et al., 2014). In addition to their antibacterial and antibiofilm properties, AMPs are also involved in modulating the immune response and anticancer activity and are termed host defense peptides (HDPs) (Raheem and Straus, 2019).

In orthopedic applications, AMPs have been utilized mainly in release modes and immobilization strategies (Costa et al., 2021). Calcium phosphate cement (CPC) is regarded as a common scaffold for the augmentation of bone defects. Our previous study reported that CPC could be applied with antibiotics for osteomyelitis treatment (Wu et al., 2021a). To overcome the resistance of methicillin-resistant *Staphylococcus aureus* (MRSA), Stallmann et al. (2003) combined CPC with human lactoferrin 1-11 (hLF1-11) AMP. The cements showed a continuous low-level release of AMP after a 24-h burst. Low concentrations of AMPs lead to biofilm dispersal, while higher doses result in biofilm cell death. In this study, AMP acted as a replacement for antibiotics in CPC, which provided sufficient bactericidal activity for resistant infections (Stallmann et al., 2003). Considering that titanium (Ti) has been widely used for orthopedic implants, the surface of Ti can be functionalized with oxhydryl and amino groups and tether biomolecules to obtain antibacterial properties (Costa et al., 2011). In one previous study, the Ti surface was amine-functionalized by a silanization process and coated with PEG to be covalently bound with enoxacin (Nie et al., 2017). To avoid systematic cytotoxicity and elongating antibacterial effects, AMPs can also be immobilized onto biomaterial surfaces and provide antimicrobial action at the implant site (Gabriel et al., 2006). FK-16, as a fragment corresponding to residues 17–32 of human LL-37, presents broad-spectrum activity against ESKAPE pathogens (Li et al., 2006). Mishra and Wang (2017) reported that Ti was coated with applied FK-16 *via* silanization treatment, which illustrated a significant inhibition of bacterial inoculation. In addition to titanium, other orthopedic implant-related polymeric and metal surfaces, such as silicone, polyethylene terephthalate, silicon, and stainless steel, can also be modified with AMPs according to an immobilization approach and possess antibacterial and antibiofilm characteristics (Costa et al., 2011).

## Nucleic Acid-Based Therapeutic Strategy Inhibits Biofilm Formation by Down-Regulating Bacterial Growth and Pathogenicity

RNA interference (RNAi) is an essential cellular regulation mechanism in which gene expression can be silenced by directly targeting a sequence (Lei et al., 2018). Accordingly, RNAi offers a new genetic medical approach targeting bone disease-associated pathogenic genes and could be a potential translational therapy for bone-related diseases (Novina and Sharp, 2004). To decrease adverse effects in non-skeletal tissues of systemic RNAi administration, bone-specific delivery systems for RNAi-based therapies are highly desirable. Zhang et al. (2015) developed a bone-targeting D-Asp8-HPMA [N-(2-hydroxypropyl) methacrylamide copolymer] polymeric nanoparticle equipped with siRNA for *sema4D* in osteoclasts. Osteoclasts can communicate with osteoblasts *via* semaphorins and suppress osteoblast maturation (Leah, 2011), which could be a promising preventative approach for infection-induced bone loss. An RNAi mechanism is illustrated in Figure 3 with a red background in the lower arrow.

Aptamers are a class of small (25–35 bases in length) single-stranded RNA (ssRNA) or DNA (ssDNA) nucleotide sequences. These specially arranged nucleotide sequences have the ability to fold into unique tertiary conformations. Based on these structures, aptamers can recognize and bind with specific targets, reminiscent of antibodies (Nimjee et al., 2005). By employing cell-SELEX, Liang et al. (2015) identified an osteoblast-specific aptamer termed CH6 (5’AGTCTGTTGGACCGAATCCCGTGGACGCACCC TTTGGACG-3’). With lipid nanoparticles encapsulating *Plekho1* siRNA, the CH6 aptamer can be specifically taken up by osteoblasts *via* micropinocytosis, which silences *the Plekho1* gene and promotes bone formation. Although there are subtle distinctions between mammalian cells and bacterial cells, nucleic acid-based strategies are also considered for bacterial targeting. In bacteria, ComE, a competence protein family, is available to recognize and transport DNA materials across bacterial cell membranes (Dubnau and Blokesch, 2019). The DNA can be recognized and taken up by the cells at an extremely fast rate, which offers a potential targeting approach on bacteria even without particular targeting moieties. As DNase is mainly located in the nucleus and mitochondria of mammalian cells, DNA-based vectors can remain stable in the cytoplasm (Boone and Tsang, 1997; Setyawati et al., 2014). While nucleases can be found on bacterial membranes, DNA degradation is triggered in transition or transformation processes (Okabayaski and Mizuno, 1974). Considering the distinct localization of DNase in mammalian and bacterial cells, DNA-based nanoparticle vectors such as tetrahedral framework nucleic acids (tFNAs) have the advantage of invading initially and combating intercellular bacterial cells such as the SCV of *S. aureus*. In a study by Setyawati et al. (2014) a DNA nanopyramid (DP) was prepared with 4 strands of oligonucleotides. Actinomycin D (AMD) was chosen as a model antimicrobial compound. By the interaction of its phenoxazone aromatic rings parallel with the guanine base of DNA, AMD is logged into the DNA framework. With the degradation of the DP structure by DNase, the released AMD could kill the infectious bacteria and then degrade the biofilms effectively.

Antisense oligonucleotides (ASOs) are short synthetic DNA or RNA molecules. These ASOs, as structural blockers that inhibit the translation of RNA, can be programmed to various sequences and specifically recognize targeting RNA by Watson–Crick base pairing. Depending on DNA/RNA heteroduplex formation, RNA cleavage is induced by RNase, and the mRNA expression level is ultimately modulated. ASOs are highly valuable as a novel strategy to treat a wide range of diseases linked to dysregulated gene expression (Quemener et al., 2022). The two-component regulatory system (TCS) commonly comprises two components termed “the sensor kinase” and “the response regulator.” TCSs are ubiquitous in bacteria but rare in mammalian cells. The membrane-bound sensor kinase mainly responds to environmental stimuli, including antibiotic stress, and induces an adaptive response with cognate response regulators, which enables bacteria to adapt to diverse environmental stresses. Therefore, TCSs have the potential to serve as targets for antimicrobial chemotherapy (Hirakawa et al., 2020). In our previous study, YycFG, also named VicRK or WalRK, was the only essential TCS contributing to bacterial pathogenicity and biofilm formation in gram-positive bacteria with a low G-C content, including *S. aureus* and *E. faecalis* (Wu et al., 2021c). Zhang et al. (2020) applied an ASO (multi-targeting the highly conserved promoter regions of *gtfBCD, gbpB*, and *ftf*, which can be modulated by VicRK TCS) to inhibit biofilm formation and *S. mutans* virulence with tFNAs as vectors. One of our studies indicated that antisense *yycF* (AS*yycF*) significantly inhibited antibiotic resistance and pathogenicity in *S. aureus* bone infection biofilms (Wu et al., 2021b). Therefore, based on the above ASO strategies and tFNA delivery systems, a TCS-targeting ASO could be developed as an efficient specific therapeutic strategy at the transcriptional level. Considering the stability of ASOs in biological fluids, many improvements have been introduced to increase resistance against nucleases. Peptide nucleic acid (PNA) molecules are the third generation of ASOs (Quemener et al., 2022). They consist of a chain of N-(2-aminoethyl) glycine units, and the nucleobases can attach to N-(2-aminoethyl) glycine units *via* a methyl carbonyl linker (Larsen et al., 1999). As a highly conserved FtsZ protein plays a role in bacterial cell division, Zhang et al. (2018) utilized an antisense PNA (asPNA) targeting the *ftsZ* gene. With tetrahedral DNA nanostructures as vectors, asPNA was successfully delivered into MRSA and effectively inhibited cell growth.

## Bacteriophage-Based Bone Infection Targeted Therapeutics Strategy to Eliminate the Not Only Planktonic but Also Biofilm Bacteria

Phages are viruses that specifically infect bacterial species. By adhering to specific surface receptors of bacteria, phages insert their hereditary material into their bacterial hosts (Onsea et al., 2020). With an estimated 10^31^ phage types (Gibb and Hadjiargyrou, 2021), the main mechanism through which phages kill bacteria is by taking over cell metabolism and ultimately inducing bacterial lysis. For *S. aureus* infection, a bacteriophage (M*^Sa^*, “M” is for mutant; “Sa” is for *S. aureus*) was identified with activity against *S. aureus*, including MRSA. M*^Sa^* can utilize the N-acetylglucosamine of the cell wall as its receptor (Gordillo Altamirano and Barr, 2021). Interestingly, *S. aureus* infected with phages can be delivered inside cells such as macrophages simultaneously with the process of *S. aureus* infecting host cells. As the phages replicate and release from infected *S. aureus*, the rest of the intracellular *S. aureus* will be subsequently affected and killed (Capparelli et al., 2007). In some cases, phages can incorporate their genome into the host genome as a prophage and reenter the lytic cycle under environmental stressors (Doss et al., 2017). This property seems to endow phages with the ability to kill persister cells in biofilms with low metabolic states, as prophages can lyse these cells when metabolic activity is recovered (Geneviere et al., 2021). This mechanism is illustrated in Figure 3 with a red background. As the resistance of bacteria in biofilms is up to 1,000-fold that of planktonic bacteria, as biofilm formation makes it difficult for conventional antibiotics to penetrate. Based on their lytic cycle, bacteriophages self-amplify in biofilms and possess an inherent ability to degrade biofilms completely (Akanda et al., 2018).

Recent evidence has shown successful bacteriophage therapy for bone and joint infections (Onsea et al., 2021). Considering the limited host range of particular bacteriophages, cocktail therapy involving more than one phage delivered simultaneously has been developed (Chan et al., 2013). When two or more phages in the cocktail attack the same bacterium, the combination may produce a better killing effect than any single phage to eliminate biofilm (Schmerer et al., 2014). In an osteomyelitis rabbit model, Kishor et al. (2016) treated MRSA infections with a cocktail of seven phages, which were selected based on their virulence. The results indicated that the phage cocktail is sufficient for treating osteomyelitis, and the combination treatment demonstrated a very good profile against infection synergistically. Yilmaz et al. (2013) combined local injection of bacteriophage Sb-1 and intraperitoneal teicoplanin administration for an MRSA implant-related infection rat model. This combination not only eradicated planktonic bacteria but also destroyed the biofilm. When phages are used to treat polymicrobial infections, multiple phages are typically applied simultaneously as a cocktail. In one previous case, a multiphage application (ᶲAbKT21phi3 and ᶲKpKT21phi1) and antibiotics (meropenem and colistin) successfully treated a patient with a trauma-related left tibial infection with XDR (extensively drug-resistant) *Acinetobacter baumannii* and MDR (multidrug-resistant) *Klebsiella pneumoniae* and avoided the serious complication of leg amputation (Nir-Paz et al., 2019).

Encapsulation of phage into a carrier is applied to control the release of phages in a musculoskeletal infection locally. Wroe et al. (2020) developed a four-arm poly (ethylene glycol)-4-maleimide (PEG-4MAL) macromer-based hydrogel that is capable of encapsulating *Pseudomonas aeruginosa* bacteriophage. In a murine local bone infection model, the antibacterial effect of the bacteriophage can remain for at least 7 days with this hydrogel (Wroe et al., 2020). Hydroxyapatite and beta-tricalcium phosphate (β-TCP) are materials commonly used in bone repair. In another study, one kind of *Escherichia coli* phage was applied to load with HA or β-TCP. This *in vitro* study demonstrated that those phages could be sustainedly released and were sufficient to eliminate the bacteria and to degrade the biofilms (Meurice et al., 2012). However, phages have varying degrees of antigenicity, although phages are not pathogens for eukaryotic cells. Notably, several mechanisms are involved in bacterial resistance to phages. Through genetic modification, genetically engineered bacteriophages can be less immunogenic and adaptive to resistance (Ly-Chatain, 2014; Gibb and Hadjiargyrou, 2021). Phage therapy was regarded as a promising strategy for persistent and resistant bacterial infections. Recent reports have described the clinical use of bacteriophages (Gelman et al., 2021). AB-SA01 (AmpliPhi Biosciences) is a manufacturing practice (GMP)-quality phage preparation involving obligately lytic tailed double-stranded DNA phages. Previously, thirteen *S. aureus* infected patients including septic shock, osteomyelitis, and infective endocarditis in Australia received the intravenous administrations of AB-SA01 as adjunctive bacteriophage therapies. There were eight patients (62%) presented clinically improved without adverse reactions even after 90 days observation, indicating the safety of bacteriophage therapy in severe *Staphylococcus aureus* infections (Petrovic Fabijan et al., 2020).

## CaP-Based Bone-Targeting Therapeutic Strategy Can Be Designed to Carry Antibiotics for Bone Infection Control

**FIGURE 4:**
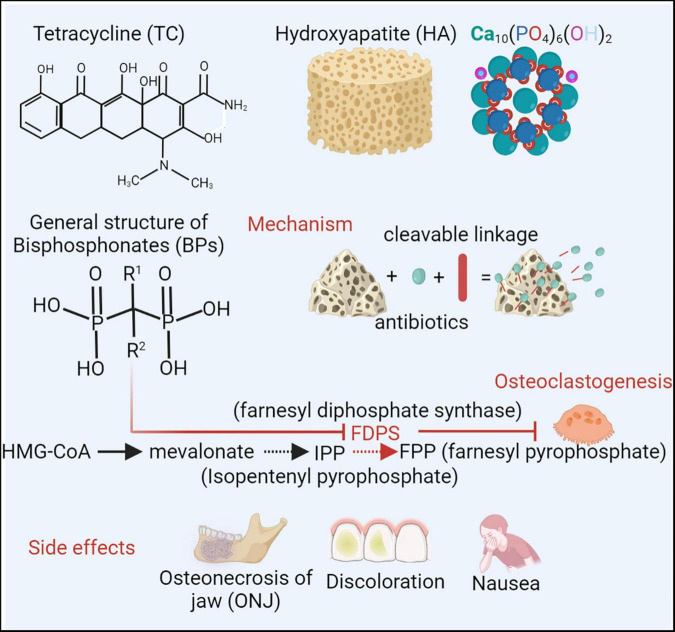
To improve therapeutic efficacy and minimize systematic toxicity, the bone targeting strategies have been applied (Shi et al., 2020). Tetracycline (TC) and its derivative are an important class of broad-spectrum antibiotics. Considering interactions with bone matrix of hydroxyapatite (HA) [Ca_10_(PO_4_)_6_(OH)_2_] and chelating with Ca^2+^, TC arouse attractions in skeletal tissues. However, side effects, such as nausea, hypoglycemia, dental discoloration, restrict TC applications as a monotherapy in osteomyelitis (Rusu and Buta, 2021). Bisphosphonates (BPs), (HO)_2_P(O)CR^1^R^2^P(O)(OH)_2_, can also interact with calcium and acquire the specificity of bone affinity. By blocking enzyme farnesyl diphosphate synthase (FDPS), BPs can inhibit FPP (farnesyl pyrophosphate) production and osteoclastogenesis, which subsequently prevents bone loss (Bjelic and Finsgar, 2022). Due to high accumulation in jawbone matrix, BP probably induce a serious complication of osteonecrosis of the jaws (ONJ) (Cheong et al., 2014). Combined with CaP based bone targeting materials, different antimicrobial agents can be conjugated for local administration. Created with BioRender.com.

CaP-based bone-targeting therapies, including tetracycline, hydroxyapatite and bisphosphonates, have a high affinity for skeletal tissues and increase the topical concentration (Figure 4). Tetracycline (TC) and its derivatives are an important class of broad-spectrum antibiotics (Shi et al., 2020; Rusu and Buta, 2021). The tetracyclic naphthacene carboxamide ring is a basic structure. Various functional groups are modified in tetracycline to create drug derivatives. TC has received particular attention in relation to skeletal tissues due to its interactions with hydroxyapatite (HA) [Ca_10_(PO_4_)_6_(OH)2] of the bone matrix by van der Waals interactions, hydrogen bonding and good metal-complexing abilities chelated with Ca^2+^ (Wang et al., 2010, 2015). Previous studies showed the anti-inflammatory and osteoblast-stimulating effects of TC, which could regulate the infective immune microenvironment and inhibit bone destruction (Reichert et al., 2012; Gomes et al., 2017). However, considering the inherent toxicity and side effects, such as nausea, hypoglycemia, tooth discoloration and increased risk of mortality, in serious infection applications, tigecycline is rarely recommended as a monotherapy in osteomyelitis (Sanchez et al., 2004; Moenster et al., 2013).

Local antimicrobial administration provides a high antibiotic concentration and reduces systemic adverse effects. This infection treatment pattern is available to eliminate biofilm persisting bacteria, such as SCVs (Huiras et al., 2012). Conventional polymethylmethacrylate (PMMA) is often utilized for the delivery of antibiotics, such as gentamicin and vancomycin, in the form of beads, which can be embedded into the surroundings of musculoskeletal infections (Cyphert et al., 2021). Due to the recurrence of *S. aureus* SCVs following local application of gentamicin in osteomyelitis, gentamicin is not recommended as a single-application treatment (Miller et al., 1978; von Eiff et al., 1997; Wright and Nair, 2010). Considering the release performance and low degradability of PMMA, a more biocompatible and bioactive calcium phosphate, hydroxyapatite (HA), has been developed as an essential element for bone formation and replacement (Lotsari et al., 2018). HA can be designed as a matrix for drug delivery, such as vancomycin in osteomyelitis (Joosten et al., 2005; Abdul Halim et al., 2021). It consists of positively charged Ca^2+^ and negatively charged PO_4_^3–^ ions, which have the ability to attract drugs as nanocarriers (Abdul Halim et al., 2021). Nanoscale particles, as a delivery system with good permeability, are intended to transport the drug to the target location in the body in a controlled manner, which reduces toxicity and side effects and relieves patients by lowering the dose (Vega-Vasquez et al., 2020). Ciprofloxacin is a broad-spectrum antimicrobial for pathogens such as *S. aureus* in long bone osteomyelitis and *P. aeruginosa* or *Aggregatibacter* spp. in jawbone osteomyelitis (Reeves et al., 2013; Kim et al., 2014). Additionally, HA can be designed as hydroxyapatite nanocomposites (HANs) to carry antibiotics such as ciprofloxacin for bone infection (Mahdavinia et al., 2019).

Bisphosphonates (BPs) are a group of compounds with the general structure (HO)_2_P(O)CR^1^R^2^P(O)(OH)_2_. The bone-specific affinity of BPs is mainly attributed to two phosphonate groups (P-C-P), which can interact with calcium to form bi- and tridentate ligands (Sun et al., 2021). BPs can also chemically link to drugs as drug-releasing conjugates. By cleavable carbamate linkage, drugs can be designed with BPs and can be released in sites under an acidic and enzymatic environment (e.g., inflammation or infection) (Ossipov, 2015). Local infection and inflammation induce accelerated bone turnover and accumulation of BPs (Sedghizadeh et al., 2017). Thus, BPs, as a bone-targeting delivery for infective treatment, are preferably targeted at skeletal sites of active metabolism, such as infection and inflammation regions. Chronic inflammation induced by infection can trigger osteoclast activation and inhibit osteoblast functioning. BPs can decrease osteoclast activation and enhance osteoblast functioning, subsequently reducing chronic inflammation. By blocking the enzyme farnesyl diphosphate synthase (FPP), BPs can inhibit osteoclastogenesis and intervene in osteoclast survival (Bjelic and Finsgar, 2022). Alendronate, a BP, has been shown to promote osteoblast activity in osteogenesis (Petrovic et al., 2020). Although BPs are regarded as a bone homeostasis regulator for bone infection therapy, there is a serious complication of osteonecrosis of the jaw (ONJ) during BP treatment, which is related to the high accumulation of BP in the jawbone matrix (Cheong et al., 2014).

## Turnover Homeostasis-Based Bone-Targeted Therapeutic Strategies on Biofilm Infection Related Immunity Homeostasis for Host-Bacteria

In addition to bacterial invasion, bone infections are accompanied by complicated immune responses with various immune cell infiltrations that release inflammatory cytokines locally and systemically (Wright and Nair, 2010). Immune cells, such as essential neutrophils and monocytes, are involved in innate or adaptive immunity against intruding pathogens and result in a severe cytokine storm, such as interleukin (IL)-1, IL-6 and TNF-α release, to produce a hyperinflammatory status. The net inflammatory reaction in the infection condition is shown in Figure 3 with a light red background. Inflammasome activation greatly contributes to bone resorption by upregulating osteoclast activity (Roper et al., 2020; Jin et al., 2021). Osteoclasts are derived from the monocyte-macrophage lineage in bone marrow and mediate bone resorption. Therefore, the combination treatment of antibiotics and immunomodulatory therapy limits the immune response and related bone destruction, which is proposed for future strategies against bone infections (Kwiecinski et al., 2013; Ali et al., 2015a,b; Jin et al., 2021). Tumor necrosis factor (TNF)–α, as a central mediator of inflammation and immune regulation, has a detrimental effect in cases of systematic *S. aureus* infections (Riegels-Nielsen et al., 1989). Fei et al. (2011) combined the TNF-α inhibitor Enbrel and antibiotics in *S. aureus* arthritis mouse models. This novel strategy significantly reduced the extent of bone damage. Additionally, the severity of staphylococcal enterotoxin shock syndrome in *S. aureus* sepsis was restricted to a low level, which may be associated with restoration of the hemostatic balance and downregulation of high-mobility group protein B1.

TNF-α inhibitors exhibit not only anti-inflammatory but also inhibitory functions in osteoclast formation. Peptidoglycan (PGN) and lipoteichoic acids (LTAs) are the major structures of the *S. aureus* cell wall (Brown et al., 2013). These structures can be recognized by the host immune system and strongly induce the release of proinflammatory cytokines, including TNF-α and IL-6. The receptor activator of nuclear factor-κB ligand (RANKL), a member of the tumor necrosis factor (TNF) superfamily, is found on the surface of osteoblasts. RANKL is active as both a trimeric transmembrane protein and a soluble monomer after it is cleaved from the cell surface by the metalloprotease-disintegrin TNF-α converting enzyme (TACE) (Kanzaki et al., 2016; Saint-Pastou Terrier and Gasque, 2017). When RANKL binds with receptor activator of nuclear factor kappa-B (RANK) on the surfaces of osteoclasts, osteoclasts can be activated, resulting in bone matrix absorption (Jin et al., 2021). In RANKL-induced osteoclastogenesis, TNF-α, produced by osteoclast progenitors, plays a mediating role (Zou et al., 2001). Hence, the combination therapy of antibiotics and anti-TNF reduces bone destruction of *S. aureus* sepsis in mice. Other inflammatory cytokine inhibitors, such as IL-1 and IL-6R antagonists, also inhibit the activation of osteoclasts and reduce inflammatory reactivity in response to pathogen-associated molecules (Tang et al., 2020). The immunomodulatory therapy is also playing an essential role in immunocompromised patients, such as patients with diabetes. In diabetic patients, the monocytes recruitment is significantly inhibited and pro-inflammatory macrophages are prevented from turning into its anti-inflammatory phenotype, which induces a prolonged inflammatory phase and leads to the chronic wound healing. The concentration of pro-inflammatory cytokines, such as tumor necrosis factor α (TNF-α) and interleukin-1b (IL-1b), are increased in diabetic wounds. Therefore, the maintenance of balanced inflammatory response is essential for the wound healing process (Rong et al., 2022). Sesamol (SM) is a natural organic compound and derived from sesame oil, which has properties including antioxidant, anti-inflammatory and antihyperglycemic. In one previous study, SM is Loaded with poly (lactic-co-glycolic acid), also named PLGA, and significantly accelerated wound process in rat diabetic foot ulcers by reduction of inflammatory mediators’ expression such as TNF-α (Gourishetti et al., 2020).

Persistent cells within biofilms can produce many virulence factors, such as *S. aureus* protein A (SpA), which is primarily anchored to the cell wall. SpA can adjust the host immunity system mainly based on the mechanism of binding with the Fc domain of antibodies. Additionally, SpA can interact with osteoblasts, preventing the expression of osteogenic markers that are essential for bone formation and mediating cell death (Claro et al., 2013). SpA can induce osteoblasts to release soluble RANKL for osteoclast migration, which promotes osteoclastogenesis and osteoclastic activity in bone resorption (Widaa et al., 2012). Accordingly, the use of the RANKL inhibitor can be a bone-targeting therapy to inhibit excessive differentiation of osteoclasts stimulated by infection-induced inflammation and limit bone destruction. Denosumab is a fully human monoclonal IgG2 antibody that inhibits bone remodeling by blocking the RANKL–RANK interaction (Kostenuik et al., 2009). Recently, denosumab has been successfully applied to suppress inflammatory joint destruction (Takeuchi et al., 2019). Compared with bisphosphonates, which are preferentially distributed in the gaps of bone resorption in trabecular bone, denosumab is more effective and distributed widely in both cortical and cancellous bone (Baron et al., 2011). However, RANKL is also expressed on immune cells (T and B cells), which can potentially inhibit immunity *via* RANKL and increase the infection rates (Ferrari-Lacraz and Ferrari, 2011).

In bone remodeling, osteoblasts can physiologically produce osteoprotegerin (OPG) to neutralize RANKL as a soluble decoy receptor and inhibit osteoclast formation and bone resorption activities (Jin et al., 2007). In *S. aureus* infection, PGN promotes osteoblast secretion of RANKL and reduces OPG production by activating the Toll-like receptor-2 pathway (Chen et al., 2014). An imbalance of these factors with an increase in the RANKL/OPG ratio is associated with increased osteoclastogenesis and results in osteolytic pathologies. In contrast to RANKL inhibitors, OPG also prevents tumor necrosis factor-related apoptosis-induced ligand (TRAIL), which plays an important role in immunosurveillance (Vitovski et al., 2007). Therefore, blockade of RANKL by administration of OPG had few effects on inflammation. Verdrengh et al. (2010) applied a RANKL-targeting treatment, OPG-Fc, in a septic arthritis mouse model. In combination with cloxacillin, the activity of osteoclasts was significantly decreased, and infection-triggered osteoporosis was limited (Verdrengh et al., 2010). Therefore, OPG may be an osteoclast-targeting agent for antibiotics.

Taken together, these targeting strategies have various potential clinical applications for biofilm and bone infections. Bone infections involving *S. aureus* and other bacterial infection is difficult to treat due to the poor penetration of antibiotics into bones and infective biofilm. The targeting antibacterial agents have been designed as an interesting strategy for infection treatment to improve the local concentrations. According to constituents of bone, CaP-based and peptide-based strategies are two main bone targeting moieties, which can be localized to enhance concentration and reduce the systemic effects. For nucleic acid-based and bacteriophage-based strategy, both moieties can be applied systemically or topically and target on specific microorganism, which facilitates the elimination of biofilm. Host immune system also plays a pivotal role in infection. Moderate inflammatory reaction can promote the infection clearance and accelerate healing process. The application of immune modulators such as TNF-α inhibitors helps to maintain the immunity homeostasis even in immunocompromised patients and promotes the infection healing.

## Conclusion

*S. aureus* is the major pathogenic bacterium for osteomyelitis, which results in a complex inflammatory response and bone destruction. Although various antibiotics have been applied widely for bone infection, the emergence of resistance and biofilm formation significantly decreases the effectiveness of those agents. To increase the local concentration and decrease unintended systemic toxicity, bone-targeting therapeutic strategies have been developed. Additionally, many peptides with repetitive acidic amino acid moieties favorably bind to the bone. By chemical conjunction with antibiotics, bone-targeting composites can release antibiotics at high local concentrations to kill bacteria effectively. Intracellular *S. aureus*, such as SCVs, particularly contributes to recurrent persisting infections. Phages can specifically infect bacterial pathogens in infected regions. Moreover, phages can infect intercellular *S. aureus* and eliminate those persister strains. CaP-based materials present a high affinity for the bone matrix *via* chelation with Ca2 + on bone. In addition to bacterial killing, imbalanced homeostasis of bone turnover is related to bone destruction. Pathogen molecule-induced cytokines can be regulated by specific antagonists and inhibit the activity of osteoclasts in bone resorption. As a supplementary strategy, immunomodulatory therapy can significantly improve antimicrobial strategies as a new approach to bone infection treatment.

## Author Contributions

SW, BW, LL, and HZ: conceptualization (equal), data curation (equal), formal analysis (equal), validation (equal), writing—original draft (equal), and writing—review and editing (equal). SD and YL: conceptualization (supporting), formal analysis (supporting), funding acquisition (lead), and writing—review and editing (equal). All authors contributed to the article and approved the submitted version.

## Conflict of Interest

The authors declare that the research was conducted in the absence of any commercial or financial relationships that could be construed as a potential conflict of interest.

## Publisher’s Note

All claims expressed in this article are solely those of the authors and do not necessarily represent those of their affiliated organizations, or those of the publisher, the editors and the reviewers. Any product that may be evaluated in this article, or claim that may be made by its manufacturer, is not guaranteed or endorsed by the publisher.
